# ﻿*Lappulaeffusa* (Boraginaceae), a new species from Xinjiang, China

**DOI:** 10.3897/phytokeys.243.123468

**Published:** 2024-06-21

**Authors:** Dan-Hui Liu, Yi-Xin Zhou, Shu-Jing Shang, Jia-Ju Wu, Wen-Jun Li

**Affiliations:** 1 State Key Laboratory of Desert and Oasis Ecology, Key Laboratory of Ecological Security and Sustainable Development in Arid Areas, Xinjiang Institute of Ecology and Geography, Chinese Academy of Sciences, Urumqi 830011, China Xinjiang Institute of Ecology and Geography, Chinese Academy of Sciences Urumqi China; 2 Conservation and Utilization of Plant Gene Resources, Key Laboratory of Xinjiang, Urumqi 830011, China Conservation and Utilization of Plant Gene Resources, Key Laboratory of Xinjiang Urumqi China; 3 College of Resources and Environment, University of Chinese Academy of Sciences, Beijing 100049, China University of Chinese Academy of Sciences Beijing China; 4 College of Forestry and Landscape Architecture, Xinjiang Agricultural University, Urumqi 830052, China Xinjiang Agricultural University Urumqi China; 5 College of Life Sciences and Technologies, Tarim University, Alar 843300, China Tarim University Alar China

**Keywords:** Boraginaceae, China, *Lappula*, new taxon, taxonomy

## Abstract

*Lappulaeffusa* D.H.Liu & W.J.Li, a new species of Boraginaceae from Xinjiang, China, is described and illustrated in this study. The new species is morphologically similar to *Lappulahimalayensis* and *L.tadshikorum*. However, it can be distinguished from the compared species by several characteristics, such as: stem single, erect, frequently branched at middle and above, densely spreading hispid, hairs discoid at base; corolla white or blue; fruit compressed, heteromorphic nutlets with two rows of marginal glochids, nutlets acute ovoid, disc narrowly ovate-triangular. The diagnosis of the new species is supported with comprehensive investigation including photographs, detailed description, notes on etymology, distribution and habitat, conservation status, as well as comparisons with morphologically similar species.

## ﻿Introduction

The genus *Lappula* Moench, belonging to the Boraginaceae family within the Rochelieae tribe, encompasses approximately with 50–70 species (Ovczinnikova 2005; Weigend et al. 2016). These species are predominantly distributed in Eurasia, North Africa, North and South America and Australia, with the centre of species diversity lying in Central Asia (Wang 1981; Ovczinnikova 2005, 2021; Huang et al. 2013). *Lappula* is characterised by prickly cauline leaves, blue/white corollas that each bear five throat appendages, a subulate gynobase, nutlets four, homomorphic or heteromorphic and nutlets with either one/more rows of marginal glochids or marginal wings tipped with anchor-like spines (Popov 1953; Riedl 1967; Zhu et al. 1995; Huang et al. 2013; Weigend et al. 2016; Khoshsokhan-Mozaffar et al. 2018).

Initially, *Lappula* had been treated as a member of *Myosotis* L. (Linnaeus 1753), with Moench (1794) later distinguishing and circumscribing *Lappula* as a separate genus. In the taxonomy of *Lappula* originating from Candolle (1846), there were 38 species in the *Prodromus* and these species were classified into three sections, based on the morphology of nutlets. In the Flora USSR, Popov (1953) identified 39 species and improved the infrageneric classification of *Lappula* by introducing two sections and 14 series. Ovczinnikova (2005) recognised 70 species and proposed an updated infrageneric classification of *Lappula*, based on corolla, nutlets and gynobase morphology. She classified the 70 species into eight sections and 14 series. Recent molecular phylogenetic studies showed that *Lappula* was polyphyletic and some species were transferred to the *Rochelia* and *Pseudolappula* (Huang et al. 2013; Khoshsokhan-Mozaffar et al. 2018; Liu et al. 2021). In China, the genus *Lappula* had 31–36 species and was highly diversified in north-western China, especially in Xinjiang Province (Wang 1981; Zhu et al. 1995).

**Figure 1.**
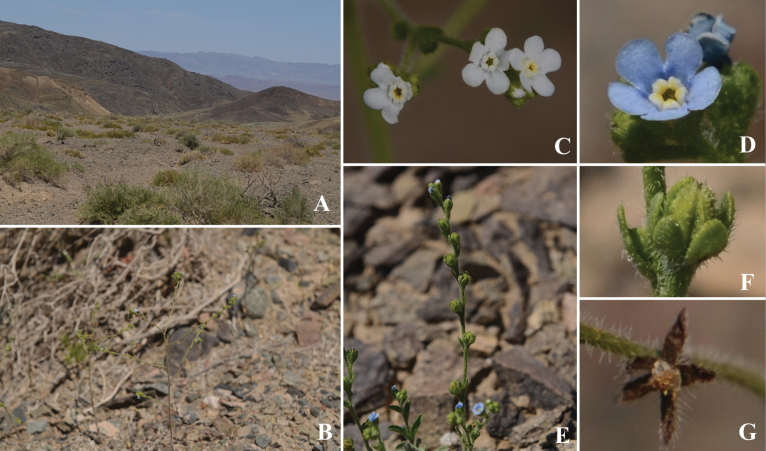
*Lappulaeffusa* D.H.Liu & W.J.Li, sp. nov. **A** habitat **B** habit **C, D** flower morphology **E** inflorescences **F** fruit **G** spreading calyx in fruit.

During field investigations in Xinjiang Province, China, an unknown population of *Lappula* was discovered in Balikun County. It appeared to be similar to *L.himalayensis* Ching J.Wang and *L.tadshikorum* Popov in general habit and fruit morphology. However, the unknown population showed great differences in an array of characters: stem single, frequently branched at middle and above, spreading; style surpassing the fruit by ca. 0.5 mm, fruit compressed, nutlets acute ovoid and disc narrowly ovate-triangular (Figs 1, 3). After conducting a comprehensive review of relevant literature (Gürke 1894; Brand 1931; Popov 1953; Vvedensky 1961; Sharashova 1962; Goloskokov 1964; Riedl 1967; Chukavina 1984; Nasir 1989; Zhu et al. 1995; Ovczinnikova 2009) and examining specimens of *Lappula* from the Herbaria of Royal Botanic Garden Edinburgh (E), Muséum National d’Histoire Naturelle (P), Komarov Botanical Institute of RAS (LE), Moscow University (MW), Central Siberian Botanical Garden SB RAS (NS), Institute of Botany, Chinese Academy of Sciences (PE), North-western Institute of Botany (WUK), Northwest Institute of Plateau Biology, Chinese Academy of Sciences (HNWP), National Herbarium of Uzbekistan (TASH) and Xinjiang Institute of Ecology and Geography, Chinese Academy of Sciences (XJBI), we concluded that it did not match morphologically with any known species of *Lappula*. Based on these distinctive morphological features, we confirmed that it was a new species, which we describe and illustrate here as *Lappulaeffusa* D.H.Liu & W.J.Li.

## ﻿Materials and methods

The voucher specimens of the new species in this study were collected during our field expedition to Xinjiang Province in 2023. Photographs were captured using a Nikon Z7 II digital camera (Tokyo, Japan), while morphological observations and measurements were conducted on living plants in the field and herbarium specimens deposited at XJBI. For morphological comparison, we critically examined available digitised specimens of *Lappula* stored in the E (https://data.rbge.org.uk/search/ herbarium/), LE (https://plant.depo.msu.ru/), MW (https://en.herbariumle.ru/), NS (http://herb.csbg.nsc.ru:8081/) and P (https://science.mnhn.fr/institution/mnhn/collection/p/item/search), as well as physical herbarium specimens deposited at PE, HNWP, TASH, WUK and XJBI. Additionally, we compared the morphological characteristics of the new species with those of similar species, relying on online or physical specimens. The conservation status was assessed following the IUCN guidelines (IUCN 2022). In this study, we employed the morphological species concept (Davis and Heywood 1963; Mallet 1995), which defines species solely by their morphological differences.

## ﻿Results

### ﻿Taxonomy

#### ﻿ Lappula effusa

Taxon classificationPlantaeBoraginalesBoraginaceae

D.H.Liu & W.J.Li sp. nov.

3F003351-4A01-5C0A-BDF5-35CC77D4FC5A

urn:lsid:ipni.org:names77343947-1

Figs 1 , 2 , 3

##### Diagnosis.

**Figure 2.**
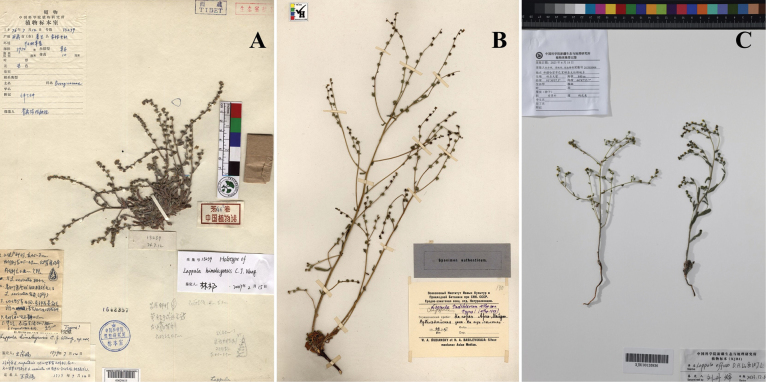
Type specimens of *L.himalayensis*, *L.tadshikorum* and *L.effusa***A** holotype of *L.himalayensis* (PE00029615!) **B** lectotype of *L.tadshikorum* (LE 140!) **C** holotype of *L.effusa* (XJBI00135936!).

**Figure 3.**
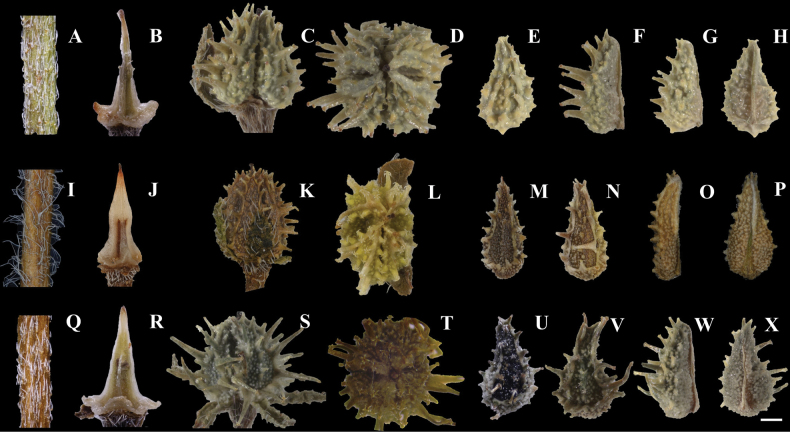
Morphological comparisons of *L.himalayensis*, *L.effusa* and *L.tadshikorum*. *L.himalayensis***A** stem indumentum **B** gynobase **C** fruit lateral view **D** fruit polar view **E** nutlet abaxial view (with short glochids) **F** nutlet lateral view (with long glochids) **G** nutlet lateral view (with short glochids) **H** nutlet adaxial view. *L.effusa***I** stem indumentum **J** gynobase **K** fruit lateral view **L** fruit polar view **M** nutlet abaxial view (with short glochids) **N** nutlet abaxial view (with long glochids) **O** nutlet lateral view **P** nutlet adaxial view. *L.tadshikorum***Q** stem indumentum **R** gynobase **S** fruit lateral view **T** fruit polar view **U** nutlet abaxial view (with short glochids) **V** nutlet abaxial view (with long glochids) **W** nutlet lateral view **X** nutlet adaxial view. Scale bar represents 0.5 mm.

The new species is morphologically similar to *Lappulahimalayensis* and *L.tadshikorum*, but differs from the *L.himalayensis* primarily in the following characteristics: stem single (vs. stems 4–6, cespitose), erect (vs. ascending or erect), frequently branched at middle and above (vs. branched above), densely spreading hispid, hairs discoid at base (vs. densely appressed pubescent); corolla white or blue (vs. blue); fruit compressed (vs. fruit globose), nutlets acute ovoid, ca. 2.5 mm long, 1 mm wide, 0.5 mm thickness (vs. ovoid, ca. 2.5 mm long, 1.5 mm wide, 1 mm thickness), the inner glochids erect (vs. often curved), disc narrowly ovate-triangular (vs. ovoid) (Figs 2, 3). Furthermore, compared to the *L.tadshikorum*, the new species is 12–28 cm tall (vs. 30–50 cm tall), stem single (vs. stems 2–3), densely spreading hair (vs. appressed or semi-appressed hair), fruit compressed (vs. fruit globose-ovoid), nutlets acute ovoid, 0.5 mm thickness (vs. ovoid, 1 mm thickness), disc narrowly ovate-triangular (vs. oblong or ovate), inner glochids erect, ca. 0.5 mm long (vs. curved, 1–1.2 mm long), style surpassing nutlets and glochids (vs. style slightly surpassing nutlets, but not surpassing glochids) (Figs 2, 3).

##### Type.

China. Xinjiang: Balikun County, Dahongliuxia Village, growing on the gravel desert, 44°47'26.17N, 91°30'9.55E, alt. 842 m, 18 June 2023, *D.H.Liu, Y.X.Zhou, S.J.Shang et al. 2023EH908* (holotype: XJBI00135936!).

##### Description.

Annual herbs. ***Stems*** erect, single, frequently branched at middle and above, 12–28 cm tall, with spreading white hispid, hairs discoid at base (Fig. 3I). ***Basal leaves*** forming a rosette; leaf blade spatulate, 1.5–2.5 cm long, 2–4 mm wide, densely spreading white hirsute, hairs discoid at base; withered in fruit; ***Stem leaves*** linear-lanceolate, 1–2 cm long, 2–4 mm wide, abaxially densely spreading white hispid, adaxially sparsely hispid or glabrous, hairs discoid at base. ***Inflorescences*** elongated to 5–10 cm long in fruit, with oval bracts near 3–5 mm long, 1.5–2.5 mm wide. ***Pedicels*** short, ca. 1 mm long in flowering and elongated from 2.5–3 mm long in fruit. ***Calyx*** lobes oblong, ca. 1.5 mm long, 1 mm wide, slightly elongated ca. 2 mm long in fruit, spread, abaxially densely spreading hispid, adaxially sparsely hispid or glabrous. ***Corolla*** blue and white (plants with either all blue or all white corollas), campanulate, corolla tube ca. 1.5 mm long; limb as long as tube, ca. 1.5 mm wide, lobes obtuse; throat appendages white or light yellow, trapeziform, ca. 0.3 mm high; stamens five, included in the corolla tube, filament short, inserted at the middle of tube, anthers brown. ***Gynobase*** narrowly subulate (Fig. 3J), with a style surpassing the nutlets by ca. 0.5 mm. ***Coenobium*** laterally compressed ovoid (Fig. 3L), with glochids 2.5–3 mm in diameter. ***Nutlets*** four, heteromorphic, easily separated from gynobase, acute ovoid; 2.5–3 mm long, ca. 1.2 mm wide, disc narrowly ovate, adaxially granulose, centre-line keeled, with a single row glochids, glochids erect; marginal glochids in 2 rows, erect, two nutlets with the inner glochids 0.5–1 mm long, outer glochids 0.2–0.5 mm long (Fig. 3N); two other nutlets with short glochids, inner glochids less than 0.5 mm long, outer glochids reduced to 0.1–0.2 mm or tuberculate (Fig. 3M); nutlets thin, ca. 0.5 mm thickness (Fig. 3O); abaxially granulose, cicatrix narrow lanceolate, ca. 1 mm long, located in the base of nutlets, adaxial keel ca. 1.5 mm long (Fig. 3P).

##### Distribution and ecology.

The new species is currently known only from its type locality in Dahongliuxia Village, Balikun County, Xinjiang Province, China. It grows in gravel desert at an elevation of 840 m above sea level.

##### Phenology.

Flowering and fruiting from May to July.

##### Etymology.

The specific epithet refers to the appearance of new species, stems frequently branched at middle and above and nearly horizontal spreading.

##### Vernacular name.

Simplified Chinese: 展枝鹤虱 (Chinese pinyin: zhǎn zhī hè shī).

##### Conservation status.

Based on the current survey data, we have only found a single population of the new species at its type locality, Dahongliuxia Village, Balikun County, Xinjiang Province, China. Data for the *Lappulaeffusa* were still insufficient to assess its conservation status. According to the IUCN Criteria (IUCN 2022), the conservation status of this new species is temporarily assessed as Data Deficient (DD) until more information becomes available.

##### Notes.

Based on the classification of *Lappula* by Ovczinnikova (2005), the new species *L.effusa* should belong to the sect. Microcarpae (M. Pop.) Ovczinnikova, ser. Tianschanicae M. Pop. ex Ovczinnikova, which is characterised by the narrowly subulate gynobase, style surpassing the nutlets by ca. 0.5–1 mm, heteromorphic nutlets with two rows of marginal glochids, disc with centre line keel. Amongst this series, there are approximately seven species (*Lappulaaktaviensis* Popov & Zakirov; *L.himalayensis*; *L.pratensis* Ching J.Wang; *L.sericata* Popov; *L.subcaespitosa* M Popov ex Golosk.; *L.tadshikorum*; *L.tianschanica* Popov & Zakirov). *L.effusa* most resembles *L.himalayensis* and *L.tadshikorum*, sharing similar corolla and gynobase morphology. However, nutlets are always important for identification and classification of *Lappula* (Popov 1953; Riedl 1967; Zhu et al. 1995; Ovczinnikova 2005) and the new species exhibits distinctive nutlet morphology from the compared species: i.e. relatively compressed fruit, acute ovoid nutlets, narrowly ovate disc and short marginal glochids. Additionally, *L.himalayensis* and *L.tadshikorum* are distributed in the mountain areas of Xizang and western Xinjiang (Himalaya and Pamir, usually 1800–4000 m a.s.l.), while *L.effusa* occurs in the gravel desert of eastern Xinjiang (lower than 1000 m a.s.l.). The detailed differences amongst these three species are provided in Table 1.

**Table 1.** Morphological comparisons of *L.effusaL.himalayensis* and *L.tadshikorum*.

Characters	*L.effusa*	*L.himalayensis*	*L.tadshikorum*
Habit	12–28 cm	7–15 cm	30–50 cm
Stem	stem single, erect, branched from middle and above, with spreading hair	stems 4–6, cespitose, ascending or erect, branched from above, with appressed hair	stems 2–3, erect, branched from above, with (semi-) appressed hair
Leave indumentum	abaxial densely spreading hispid, adaxially sparsely hispid or glabrous	abaxial densely appressed pubescent, adaxially sparsely pubescent	abaxial densely spreading hair, adaxially sparsely
Inflorescences	5–10 cm in fruit	3–7 cm in fruit	3–7 cm in fruit
Pedicel	2–3 mm long in fruit	1.5–2 mm long in fruit	1–3 mm long in fruit
Calyx	ca. 2 mm and shorter than the nutlets	ca. 2 mm and shorter than the nutlets	ca. 2 mm and shorter than the nutlets
Corolla	blue or white	blue	blue
Style	surpassing the fruit ca. 0.5 mm and surpassing glochids	surpassing the fruit ca. 0.5 mm and surpassing glochids	surpassing the fruit, but not surpassing glochids
Nutlets	heteromorphic; acute ovoid	heteromorphic; ovoid	heteromorphic; ovoid
Marginal glochids	two rows; 0.5–1 mm long inner glochids erect	two rows; 1.5 mm long; inner glochids often curved	two rows; 1–1.2 mm long; inner glochids curved
Disc of nutlets	narrowly ovate-triangular	ovoid	ovoid
Flowering and fruiting	May to July	June to August	June to July
Elevation	840 m	3700–4200 m	1800–3000 m

## Supplementary Material

XML Treatment for Lappula effusa

## ﻿Additional information

### Author ORCIDs

Dan-Hui Liu https://orcid.org/0000-0002-0195-1436

Yi-Xin Zhou https://orcid.org/0000-0002-0054-0301

Shu-Jing Shang https://orcid.org/0009-0006-8092-7616

Jia-Ju Wu https://orcid.org/0009-0009-0640-0754

Wen-Jun Li https://orcid.org/0000-0002-2932-0783

### Data availability

All of the data that support the findings of this study are available in the main text.
